# What’s behind the white coat: Potential mechanisms of physician-attributable variation in critical care

**DOI:** 10.1371/journal.pone.0216418

**Published:** 2019-05-16

**Authors:** Kuldeep N. Yadav, Michael Josephs, Nicole B. Gabler, Michael E. Detsky, Scott D. Halpern, Joanna L. Hart

**Affiliations:** 1 Palliative and Advanced Illness Research Center, University of Pennsylvania, Philadelphia, Pennsylvania, United States of America; 2 Fostering Improvement in End-of-Life Decision Science Program, University of Pennsylvania, Philadelphia, Pennsylvania, United States of America; 3 Center for Health Incentives and Behavioral Economics, University of Pennsylvania, Philadelphia, Pennsylvania, United States of America; 4 Center for Clinical Epidemiology and Biostatistics, University of Pennsylvania, Philadelphia, Pennsylvania, United States of America; 5 Division of Critical Care Medicine, UHN/Mount Sinai Hospital, Toronto, Canada; 6 Department of Medicine, University of Toronto, Toronto, Canada; 7 Leonard Davis Institute of Health Economics, University of Pennsylvania, Philadelphia, Pennsylvania, United States of America; 8 Division of Pulmonary, Allergy, and Critical Care Medicine, University of Pennsylvania, Philadelphia, Pennsylvania, United States of America; 9 Department of Medical Ethics and Health Policy, University of Pennsylvania, Philadelphia, Pennsylvania, United States of America; University of Notre Dame Australia, AUSTRALIA

## Abstract

**Background:**

Critical care intensity is known to vary across regions and centers, yet the mechanisms remain unidentified. Physician behaviors have been implicated in the variability of intensive care near the end of life, but physician characteristics that may underlie this association have not been determined.

**Purpose:**

We sought to identify behavioral attributes that vary among intensivists to generate hypotheses for mechanisms of intensivist-attributable variation in critical care delivery.

**Methods:**

We administered a questionnaire to intensivists who participated in a prior cohort study in which intensivists made prognostic estimates. We evaluated the degree to which scores on six attribute measures varied across intensivists. Measures were selected for their relevance to preference-sensitive critical care: a modified End-of-Life Preferences (EOLP) scale, Life Orientation Test–Revised (LOT-R), Jefferson Scale of Empathy (JSE), Physicians' Reactions to Uncertainty (PRU) scale, Collett-Lester Fear of Death (CLFOD) scale, and a test of omission bias. We conducted regression analyses assessing relationships between intensivists’ attribute scores and their prognostic accuracy, as physicians’ prognostic accuracy may influence preference-sensitive decisions.

**Results:**

20 of 25 eligible intensivists (80%) completed the questionnaire. Intensivists’ scores on the EOLP, LOT-R, PRU, CLFOD, and omission bias measures varied considerably, while their responses on the JSE scale did not. There were no consistent associations between attribute scores and prognostic accuracy.

**Conclusions:**

Intensivists vary in feasibly measurable attributes relevant to preference-sensitive critical care delivery. These attributes represent candidates for future research aimed at identifying mechanisms of clinician-attributable variation in critical care and developing effective interventions to reduce undue variation.

## Introduction

Critical care intensity varies across geographic regions and health care centers [1–5]. Most of this variation is not explained by patients’ characteristics or preferences [1, 6–9], nor by measured center-level characteristics such as academic affiliation [10–12]. However, preliminary evidence suggests that physician identity comprised of individual characteristics and personal beliefs may be a potential source of this variation [13–15]. For example, physician identity has been shown to more strongly predict patient enrollment in hospice care than patient characteristics [10]. Such physician-attributable variation of care that should be based on patient preferences, values, and goals is inherently problematic. Reducing physician-attributable variation in preference-sensitive health care choices is paramount, yet the mechanisms of such variation remain unclear. Identifying these mechanisms is the first step in a research agenda aimed at developing interventions to reduce this undue variation.

The frequency and complexity of critical care decision making [16], including preference-sensitive decisions to use or limit life-extending therapies [17], provides an ideal context to identify mechanisms of physician-attributable variation [18]. Furthermore, this context largely eliminates the possibility of confounding by patient preferences because patients are typically assigned a critical care physician, or intensivist, based solely on physician scheduling [19].

Thus, as a first step toward identifying and understanding the mechanisms underlying individual physicians’ approaches to treatment intensity, we sought to identify attributes that are feasible for use among physicians, differ across intensivists, and may influence their individual approaches to clinical decision making (e.g., prognostication). We reasoned that only characteristics that varied considerably across intensivists would warrant future investigation for causal contributions to practice variability.

## Methods

We aimed to identify attributes that varied across intensivists who contributed to a previously published cohort study [20]. This population was selected because these intensivists were already known to vary in a clinical skill, specifically their prognostic abilities. Furthermore, prognostic accuracy may be one manner by which individual physicians influence preference-sensitive decisions. Thus, we only surveyed intensivists who provided prognostic estimates on at least five critically ill patients requiring life support in order to ensure a reasonable number of predictions for comparing physicians’ prognostic accuracy and behavioral attributes (S1 Fig). Roughly 1 year after their final prediction, we sent eligible intensivists an online survey consisting of a demographics questionnaire, five attribute scales, and an omission bias test (S2 Fig). Informed consent was explicitly obtained and documented from all individual participants prior to their participation, and all aspects of this study was approved by the institutional review board of the University of Pennsylvania.

For this exploratory study, we selected psychological attributes that were previously validated, easy to measure, and particularly relevant to preference-sensitive critical care delivery. These six attributes included (A) preferences for end-of-life care, (B) optimism, (C) empathy, (D) reaction to uncertainty, (E) fear of death, and (F) susceptibility to the omission bias. We used six standardized instruments to measure the attributes: (A) End-of-Life Preferences (EOLP) scale, a 22-item measure of comfort-oriented versus life-sustaining treatment preferences in the event of neurologic or physical impairment [21]; (B) Life Orientation Test-Revised (LOT-R), a 6-item measure of dispositional optimism that may explain differences in physicians’ interpretation and communication of prognostic information during critical illness [22]; (C) Jefferson Scale of Empathy (JSE) Physician Version, a 20-item measure of empathy [23]; (D) Physicians' Reactions to Uncertainty (PRU), a 22-item measure of the stress from and reluctance to disclose medical uncertainty [24]; (E) Collett-Lester Fear of Death (CLFOD) scale, a 28-item measure of the concerns about death and dying that may influence the provision of comfort-oriented care [25]; and (F) a modified omission bias test [26], which assesses physicians’ susceptibility to the omission bias and preference for harmful inaction. A response greater than 50% on the omission bias test (n = 1) was discarded because it was undistinguishable whether the respondent misunderstood the question or actually displayed an opposite bias towards harmful action.

We used calculated prognostic accuracy scores for each physician in our cohort in order to assess for associations between this variable clinical skill and the newly measured attributes [20]. These scores were their overall proportions of correct predictions for patients’ (A) in-hospital survival and outcomes assessed six months following ICU admission: (B) six-month survival, (C) return to original residence, and abilities to (D) ambulate 10 steps and (E) toilet independently, or (F) have normal cognition as prior to ICU admission.

We described the distributions of responses to each instrument and examined correlations among attributes using Spearman rank correlation tests. When applicable, we assessed differences between instrument subscale scores using Wilcoxon signed-rank tests. We used bivariate regression to assess relationships between the intensivists’ attribute scores and prognostic accuracy scores. For all analyses, p-values <0.05 were considered statistically significant. We did not attempt multivariable modeling due to the inherent limited degrees of freedom available in this hypothesis-generating study. Statistical analyses were conducted in Stata (v15.0, StataCorp, College Station, TX) and R Studio (v1.0.153, RStudio Inc., Boston, MA) using the R language for statistical computing (v3.4.1, R Foundation, Vienna, Austria) and the R package ggplot2 (v2.2.1, Springer-Verlag, New York, NY).

## Results

20 of 25 (80%) eligible intensivists completed the survey. The majority identified as male (90%), White (92%), and non-Hispanic (96%). The participants otherwise represented a diverse group of intensivists in terms of age, primary specialty, and proportion of professional time spent providing clinical critical care (Table 1).

**Table 1:** Intensivists’ characteristics.

Characteristic (n = 20)	Number (%)
Age*	
40 or less	6 (30%)
41–50	5 (25%)
51–60	5 (25%)
61–70	3 (15%)
**Gender**	
Male	18 (90%)
Female	2 (10%)
**Race**	
White	18 (90%)
Non-white	2 (10%)
**Ethnicity**	
Hispanic	1 (5%)
Non-Hispanic	19 (95%)
**Medical specialty**	
Internal Medicine	15 (75%)
Anesthesia	3 (15%)
Surgery	2 (10%)
**Years as attending intensivist**	
1–5	4 (20%)
6–10	6 (30%)
11–20	3 (15%)
21 or more	7 (35%)
**Percentage of professional time spent providing critical care**	
20% or less	10 (50%)
21–40%	6 (30%)
41% or greater	4 (20%)

*1 physician with missing information.

**Fig 1:**
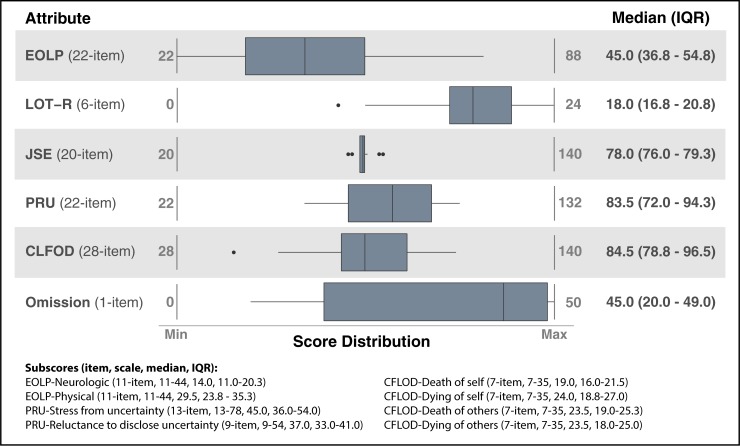
Median and IQR of intensivists’ attribute scores. **Definition of abbreviations:** EOLP = modified End-of-Life Preferences scale, LOT-R = Life Orientation Test-Revised, JSE = Jefferson Scale of Empathy, CLFOD = Collett-Lester Fear of Death Scale, PRU = Physicians' Reactions to Uncertainty Scale, Omission = modified omission bias measure. **Direction of scores:** Higher EOLP scores indicate a preference for aggressive treatment over comfort-oriented treatment. Higher LOT-R scores suggest greater dispositional optimism. Higher JSE scores indicate a more empathic behavioral orientation. Higher PRU scores suggest greater stress from and reluctance to disclose medical uncertainty. Higher CLFOD scores suggest greater fear of death and dying. Lower Omission scores suggest greater susceptibility to omission bias.

Intensivists varied in their personal end-of-life preferences, dispositional optimism, reactions to uncertainty, fear of death and dying, and susceptibility to omission bias. However, they demonstrated little variability in empathy (Fig 1). None of the attributes were strongly correlated with each another (all r_s_<0.4, all *p*>0.05, S3 Fig).

Despite variability in end-of-life care preferences, most intensivists preferred comfort-oriented care in the event of a catastrophic illness with stronger preferences for comfort-oriented care in the event of neurologic rather than physical impairment (p<0.001). Intensivists were generally more optimistic than pessimistic but varied in their degree of optimism. Intensivists’ empathy scores tended to be lower than those of other medical specialties [27], but demonstrated minimal within-sample variation. Intensivists displayed discomfort and apprehension regarding medical uncertainty and fear of death and dying, with substantial variation in the degree to which they experience such distress. The majority of intensivists demonstrated some susceptibility to the omission bias, thereby suggesting a slight preference for harmful inaction. Intensivists’ median prognostic accuracy scores were ≥70% for five domains with variation in all domains (S4 Fig). Additionally, prognostic accuracy for in-hospital and six-month survival increased by 5% and 4%, respectively, for every one point increase in EOLP scores favoring aggressive treatment (Table 2). There were identified associations between intensivists’ EOLP scores and their accuracy of predicting in-hospital and six-month survival (both *p*<0.05).

**Table 2:** Association between intensivists’ attributes and prognostic accuracy.

		Prognostic Accuracy					
		In-hospital Survival	Six-month Survival	Return to original residence	Ambulate 10 steps independently	Toileting independently	Normal cognition
		β coef (95% CI)	β coef (95% CI)	β coef (95% CI)	β coef (95% CI)	β coef (95% CI)	β coef (95% CI)
**Attributes**	**EOLP**	0.05* (0.01–0.08)	0.04* (0.02–0.05)	0.00 (-0.04–0.03)	0.02 (-0.01–0.04)	0.00 (-0.03–0.03)	0.01 (-0.01–0.03)
	**LOT-R**	0.10 (-0.03–0.23)	0.05 (-0.06–0.15)	0.03 (-0.07–0.14)	0.00 (-0.10–0.10)	0.05 (-0.02–0.12)	0.03 (-0.05–0.11)
	**JSE**	0.00 (-0.17–0.16)	0.01 (-0.06–0.08)	-0.06 (-0.17–0.04)	-0.07 (-0.17–0.03)	-0.03 (-0.17–0.12)	-0.01 (-0.22–0.21)
	**PRU**	-0.01 (-0.07–0.05)	0.00 (-0.03–0.03)	-0.02 (-0.06–0.02)	-0.01 (-0.04–0.03)	-0.01 (-0.05–0.03)	-0.01 (-0.04–0.03)
	**CLFOD**	0.01 (-0.02–0.03)	0.00 (-0.02–0.02)	0.01 (-0.02–0.03)	0.00 (-0.02–0.02)	0.00 (-0.02–0.02)	-0.02 (-0.04–0.01)
	**Omission**	0.01 (-0.02–0.05)	0.00 (-0.02–0.01)	0.00 (-0.02–0.02)	0.01 (-0.02–0.03)	0.00 (-0.03–0.02)	-0.01 (-0.03–0.02)

*p < 0.05

Definition of abbreviations: EOLP = modified End-of-Life Preferences scale, LOT-R = Life Orientation Test-Revised, JSE = Jefferson Scale of Empathy, CLFOD = Collett-Lester Fear of Death Scale, PRU = Physicians' Reactions to Uncertainty Scale, Omission = modified omission bias measure

Direction of scores: Higher EOLP scores indicate a preference for aggressive treatment over comfort-oriented treatment. Higher LOT-R scores suggest greater dispositional optimism. Higher JSE scores indicate a more empathic behavioral orientation. Higher PRU scores suggest greater stress from and reluctance to disclose medical uncertainty. Higher CLFOD scores suggest greater fear of death and dying. Lower Omission scores suggest greater susceptibility to omission bias.

## Discussion

This study reveals variability across intensivists in several validated, measurable attributes that may influence how physicians care for and communicate with patients and their surrogate decision makers. These identified behavioral and decision-making qualities may explain physician-level differences in preference-sensitive critical care delivery, a phenomenon for which explanatory mechanisms remain elusive [28–30]. Variation in these characteristics is necessary, but not sufficient, to implicate these characteristics in mechanisms underlying such variation. Thus, these data provide guidance regarding which measures to select in larger studies seeking to establish the mechanisms of physician-attributable variation and to develop interventions aimed at reducing undue variation.

This study has several limitations. First, the small sample yielded limited powered to detect associations between intensivists’ attributes and prognostic accuracy scores. Consequently, multivariable modeling was not possible. While our exploratory analyses did yield some associations between physicians’ attributes and prognostic accuracy, these associations may be due to Type 1 error. Therefore, further investigation is needed in order to determine whether any of the six personal attributes of physicians actually influence their clinical predications. Second, sampling intensivists from a single health care system may limit the generalizability of the findings. However, the presence of physician-level variation in this single sample suggests that such variation is also likely in a more diverse population. Third, physicians’ attributes at the time of prognostication may not be consistent with their attributes approximately one year later at the time of survey completion, which may have contributed to our null regressions. However, evidence regarding the temporal stability of these attributes is lacking. Finally, although we selected attributes which we hypothesized would contribute to variation in clinical practice, additional attributes that we did not measure may vary among intensivists.

Nonetheless, this study provides a formative step in a research agenda that aims to reduce problematic physician-attributable variation in critical care by identifying behavioral and decision-making attributes that vary among intensivists. These attributes will be important tools in future work that elucidate the mechanisms of physician-attributable variation in a larger sample, including examining the relationship between physicians’ attributes and patterns of care intensity and patient outcomes. Understanding the role and causes of variation due to physicians is a necessary first step in developing and testing communication and decision-making interventions that better align the care patients receive with their own goals and preferences.

## Supporting information

S1 Fig Flow diagram depicting the participation of intensivists. (EPS)

S2 Fig Omission bias test. (DOCX)

S3 Fig Spearman rank correlation coefficients (*r*_*s*_) denoting association among intensivists’ attributes. (EPS)

S4 Fig Median and IQR of intensivists’ prognostic accuracy scores. (EPS)

S1 Data Excel spreadsheets containing the minimal dataset and data dictionary. (XLSX)
