# Supplementary material for: What’s behind the white coat: Potential mechanisms of physician-attributable variation in critical care
Source: PLoS One. 2019 May 16;14(5):e0216418. doi: 10.1371/journal.pone.0216418 (PMC6522043; doi:10.1371/journal.pone.0216418)
Supplement: S2 Fig — (DOCX) [file pone.0216418.s002.docx]

**S2 Fig. Omission Bias Test.**

Clinicians in the ICU work hard to prevent death from serious illnesses when possible. Imagine there is a new treatment for an ICU illness for which no other treatments are available. This treatment has been studied with several high-quality randomized trials and is available for use at no cost. You are taking care of a patient with the illness and must decide whether to recommend the treatment.

Without treatment, some patients will die from the illness before leaving the hospital. The treatment is always effective when it is used, meaning that no one who receives the treatment will die from the disease. However, the treatment itself may result in some patients dying from the treatment's side effects. The side effects do not otherwise increase pain or suffering.

1. *If 50% of patients with the illness die without treatment and 5% of patients who receive the treatment die, would you recommend the treatment?*

yes | no (Please select one)

1. *If 50% of patients with the illness die without treatment and 95% of patients who receive the treatment die, would you recommend the treatment?*

yes | no (Please select one)

1. *If 50% of patients with the illness die without treatment, what is the highest proportion (%) of treatment-related deaths that you would accept in order to recommend the treatment to a patient with the illness?*

_______ (Please provide an integer from 0 to 100)

Notes for scoring question 3:

- Discard any response(s) greater than 50% because it is undistinguishable whether the respondent(s) misunderstood the question or actually displayed an opposite bias towards harmful action.
- The lower Omission score, the greater susceptibility to the omission bias and preference for harmful inaction.
